# Deletion of protein kinase C θ attenuates hepatic ischemia/reperfusion injury and further elucidates its mechanism in pathophysiology

**DOI:** 10.22038/ijbms.2024.77365.16730

**Published:** 2024

**Authors:** Wei Li, Meng-Yuan Shen, Ruo-Bing Liu, Jun-Yang Zhang, Rong-Yu Li, Guo-Guang Wang

**Affiliations:** 1 Department of Pathophysiology, Wannan Medical College, Wuhu, China; 2 School of Medical Imaging, Wannan Medical College, Wuhu, China; 3 School of Clinical Medicine, Wannan Medical College, Wuhu, China; 4 Department of Immunology, Wannan Medical College, Wuhu, China

**Keywords:** Gene knockout, Hepatic ischemia/reperfusion injury, Nrf2/HO-1 pathway, Pathophysiology, Protein kinase C θ, TLR4/NF-κB/IKB α pathway

## Abstract

**Objective(s):**

Hepatic ischemia-reperfusion (HIR) is a severe process in pathophysiology that occurs clinically in hepatectomy, and hepatic transplantations. The present study aimed to investigate the effect of PKC θ deletion against HIR injury and elucidate its mechanism in pathophysiology.

**Materials and Methods:**

HIR injury was induced in wild-type and PKC θ deletion mice treated with or without heme. The ALT and AST levels were determined to evaluate liver function. HIR injury was observed via histological examination. Oxidative stress and inflammatory response markers, and their signaling pathways were detected.

**Results:**

The study found that PKC θ knockout decreased serum AST and ALT levels when compared to the WT mice. Furthermore, heme treatment significantly reduced the ALT and AST levels of the PKC θ deletion mice compared with the untreated PKC θ deletion mice. PKC θ deletion markedly elevated superoxide dismutase activity in the liver tissue, reduced malondialdehyde content in the tissue, and the serum TNF-α and IL-6 levels compared with the WT mice. Heme treatment was observed to elevate the activity of SOD and reduced MDA content and serum of TNF-α and IL 6 in the PKC θ deletion animals. Meanwhile, heme treatment increased HO-1 and Nrf 2 protein expression, and reduced the levels of TLR4, phosphorylated NF-κB, and IKB-α.

**Conclusion:**

These findings suggested that PKC θ deletion ameliorates HIR, and heme treatment further improves HIR, which is related to regulation of PKC θ deletion on Nrf 2/HO-1 and TLR4/NF-κB/IKB α pathway.

## Introduction

Hepatic ischemia/reperfusion (HIR) results from blood deprivation of the liver following restoration of blood flow. During IR, a shortage of oxygen and nutrient supply caused by ischemia leads to tissue and cell damage, and subsequent restoration of blood perfusion results in more serious damage to tissue and cells. Hepatic IR injury is a complex pathophysiological process that occurs clinically in hepatectomy, hepatic transplantations, liver trauma surgery, and resuscitation from shock (1, 2). Although the mechanism responsible for hepatic IR injury is not yet elucidated, several factors and signaling systems have been demonstrated to play an important role in the injury. Some studies have demonstrated that hepatic ischemia and reperfusion increase the generation of reactive oxygen species (ROS) and proinflammatory factors, including tumor necrosis factor (TNF)-α and interleukin (IL)-1 via the Toll-like receptor (TLR)4/ nuclear factor kappa B (NF-**κB**) pathways, contributing to the progress of hepatic ischemia/reperfusion injury (3-6).

Protein kinase C (PKC), a group of protein kinases that are characterized by phospholipid-dependence and serine/threonine, includes various isozymes that are grouped into three subfamilies due to their different feature: conventional or classic PKC isozymes (cPKCs) such as PKC **α** and γ, novel or non-classic PKC isozymes (nPKCs) such as PKC θ, η, and δ, and atypical PKC isozymes (aPKCs) including PKC λ, ι and ζ (7, 8). PKCs play important roles in multiple signal pathways and are implicated in cell differentiation, proliferation, migration, regulation of gene expression, extracellular matrix proteins, and apoptosis (9, 10). PKCs are confirmed to be involved in various pathophysiological processes, including Alzheimer’s disease, cardiovascular diseases, and cancers (8, 9). Further evidence showed that inhibition of some PKC isozymes exerts protective effects on ischemia/reperfusion injury, PKC ζ and δ inhibition attenuate cardiac and brain ischemia/reperfusion injury, respectively (11).

PKC θ, one of PKC isozymes, is expressed in T-cells, skeletal muscle cells, and platelets. PKC θ exerts pivotal roles in several inflammatory diseases through modulation of T-cell activation such as allergic lung inflammation, and multiple sclerosis. (12). Further studies showed that inhibition or down-regulation of PKC θ improves regeneration of muscle stem cells, anti-asthmatic activity, and ulcerative colitis (13-15). However, our colleagues and students wondered if and how PKC θ is involved in the pathophysiological processes of hepatic ischemia/reperfusion injury, and how PKC θ regulates hepatic ischemia/reperfusion injury in pathophysiology. PKC θ deficient mice were often used to illustrate the mechanism of PKC θ in the pathogenesis of disease (16).

To make our colleagues and students understand the effect of PKC θ in ischemia/reperfusion injury, this study aims to investigate the mechanism of PKC θ in hepatic ischemia/reperfusion injury in PKC θ gene knockout mice.

## Materials and Methods

***Materials***

Sodium pentobarbital and heme were the products of Sigma (St Louis, USA). Specific-TNF-α and IL-6 ELISA kits were products of Hefei Bomei Biotechnology CO., LTD, (Hefei, China). Assay kits of MDA and SOD detection were obtained from Nanjing Jiancheng Bioengineering Institute (Nanjing, China). Antibodies used in this experiment were purchased from Cell Signaling Technology Inc (Boston, USA). IgG goat anti-rabbit secondary antibodies were obtained from Wuhan Boster Biological Technology, Ltd (Wuhan, China).

***Animals and the study protocol***

PKC θ knockout C57BL/6 mice were purchased from Shanghai Genechem Co., LTD (Shanghai, China). Wild-type (WT) mice were obtained from Changsha Tianqin Biotechnology Co., Ltd (Changsha, China). An animal model of Hepatic ischemia/reperfusion (I/R) injury was implemented as reported in previous research (17). Firstly, the mice were anesthetized with pentobarbital sodium (IP, 45 mg/kg), and an incision in the midline of the abdomen was performed to open the abdomen. The conjunct blood vessel towards the left and middle liver lobes was separated and clamped using an atraumatic clip to establish a liver ischemia model. After 1 hr of ischemia, the clip was removed for a reperfusion period of 6 hr. To explore the mechanisms of the effect of PKC θ deletion on liver ischemia/reperfusion injury, PKC θ knockout and WT mice were treated with heme (20 μg/kg) once to induce heme oxygenase-1 (HO-1) before a week of liver ischemia/ reperfusion according to aforementioned methods (18). 

***Sample preparation***

After the reperfusion period of 6 hr, blood was obtained, and centrifugation of 1000 g for 10 min was performed to separate the serum. The serum was used to determine alanine aminotransferase (ALT), aspartate aminotransferase (AST), and cytokines. Part of the left liver was obtained and fixed in the 10% neutral formalin for histopathological examinations. Other liver samples were collected and stored at 80 ^°^C for western blot analysis.

***Liver function analysis***

To assess liver function, AST and ALT levels in serum were determined with an automated biochemical analyzer.

***Assessment of oxidative stress***

The liver was homogenized and lysed in phosphate-buffered saline (PBS, pH 7.4) on ice. The lysis solution was separated via centrifugation of 1000 g for 15 min at 4 ^°^C. The supernatant was collected and used to detect antioxidant enzyme SOD activity and the lipid peroxidation product malondialdehyde (MDA) content for assessment of oxidative stress in the liver. SOD activity and MDA content were determined using biodiagnostic assay kits.

***Inflammatory response analysis***

Serum inflammatory factors, such as NF-**α** and IL-6 were measured with specific- NF-α and IL-6 ELISA kits according to the manufacturer’s protocol.

***Assessment of histology***

Fixed liver tissues were dehydrated and embedded in paraffin. Liver tissue sections were cut into 5-μm thickness and stained with hematoxylin for 5 min and subsequently eosin for 3 min at 25 ^°^C. Morphometric features of the liver were observed under a light microscope.

***Immunofluorescence assay***

After rinsing 3 times with PBS-T, liver tissue sections were incubated with 5% horse serum to block non-specific sites. Furthermore, the sections were incubated with the following specific antibodies: Caspase 3 and NF-κB primary antibodies (1:100) overnight at 4 ^°^C. After being washed 3 times with PBS, the sections were incubated with FITC conjugated secondary antibody (1:500)(Biosharp, Hefei, China). The sections were analyzed with a laser confocal TCSSP8 microscope.

***Western blot***

Mice liver tissues were lysed in ice-cooled homogenization buffer (20 mmol/l Tris, 1 mmol/l EDTA, 2 mmol/l EGTA, 150 mmol/l sodium chloride) containing 2 mmol/l PMSF, 2 μg/ml leupeptin, and 2 μg/ml aprotinin. The homogenate was centrifugated at 12000 g for 15 min at 4 ^°^C. Protein in the supernatants was determined with a BCA kit according to the manufacturer’s instruction. Protein in the supernatants was separated using sodium dodecyl-sulfate polyacrylamide gel electrophoresis and then electrophoretically transferred to PVDF membranes. The membranes were immersed into 5% skimmed milk containing the following specific antibodies: β-actin, HO1, Nrf 2, IKB**α**, p- IKB**α**, NF-κB, and pNF-κB overnight at 4 ^°^C, respectively. After rinsing with PBS, the membranes were incubated with HRPconjugated goat antirabbit IgG secondary antibodies. Target protein was shown using the chemiluminescence detection kit (Beijing labgic Biotechnology CO., LTD. Beijing, China) in accordance with the manufacturer’s protocol.

***Statistical analysis***

The experimental data are expressed as means ± standard deviation (SD). Statistical differences between groups were carried out by an unpaired Student’s t-test or one-way analysis of variance (ANOVA) and corrected using a Bonferroni/Dunn test. GraphPad Prism (Version 7.00) was used to process the experimental data. *P*<0.05 was considered statistically significant.

## Results

***Features of ischemia/reperfusion***

**Figure 1:**
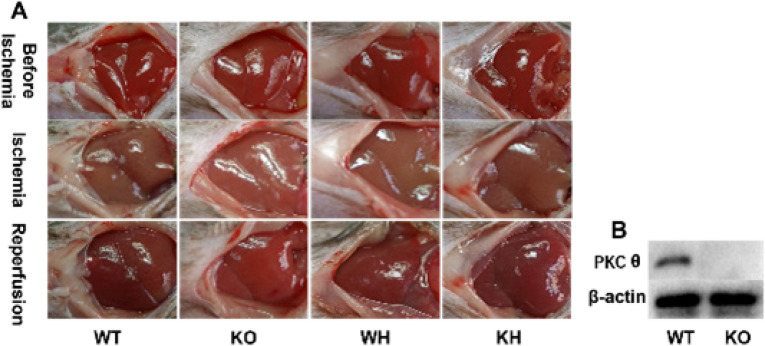
Feature of hepatic ischemia/reperfusion injury. (A) Feature of hepatic ischemia/reperfusion injury. (B) Expression of PKC θ protein in the liver

WT: wild-type mice; KO: PKC-θ knockout mice, WH: wild-type mice treated with heme; KH: PKC-θ knockout mice treated with heme

To elucidate the pathophysiological role of PKC θ in hepatic ischemia/reperfusion (HIR) injury, a model of HIR injury was prepared in PKC θ deletion mice. Therefore, the expression of PKC θ protein was detected in the mice, the results showed that PKC θ protein expression was not observed in the PKC θ deletion mice (Figure 1B).

Before ischemia, the livers are red. The livers gradually became grey after ischemia, and crimson following reperfusion (Figure 1A).

***Changes in liver function markers***

**Figure 2:**
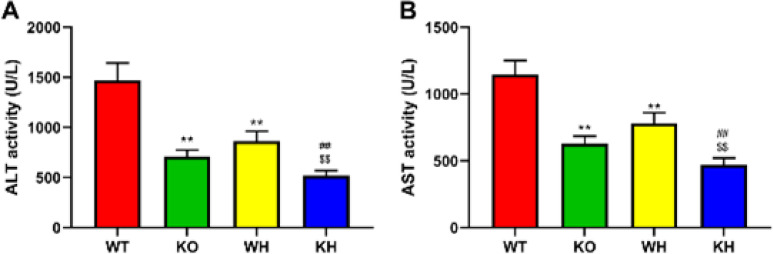
Changes of hepatic function markers. (A) ALT activity. (B) AST activity

In this study, we trialed whether PKC θ knockout protected against HIR injury. The PKC θ deletion significantly reduced the markers of liver function such as serum ALT and AST in the HIR PKC θ deletion when compared to the HIR WT mice (Figure 2). Furthermore, to explore the mechanism of PKC θ deletion the pathophysiology of the HIR injury was explored, therefore induction of heme oxygenase by heme in the HIR mice was used to investigate the changes of the HIR injury. Heme was observed to significantly reduce the serum levels of markers of liver function in the HIR PKC θ deletion or WT mice when compared to untreated PKC θ knockout or WT mice (Figure 2), respectively. Additionally, the serum levels of markers of liver function were decreased in the heme-treated HIR PKC θ deletion mice compared with the heme-treated WT mice (Figure 2).

***Assessment of antioxidative activities***

**Figure 3:**
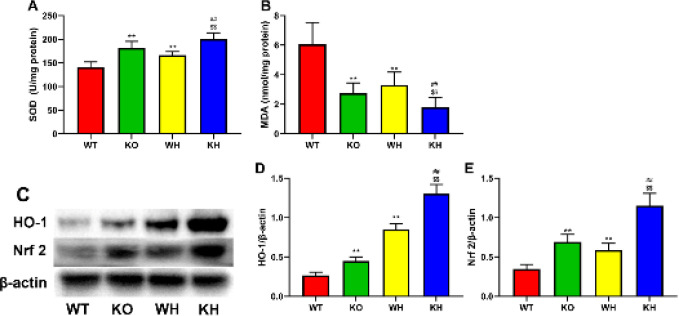
Effect of PKC-θ knockout on oxidative stress. (A) SOD activity in the liver. (B) MDA level in the liver. (C) Expression of HO-1 and Nrf 2 in the liver. (D) Relative level of HO-1. (E) Relative level of Nrf 2

To detect the effect of PKC θ deletion on oxidative stress, the lipid peroxidation and oxidative stress marker malondialdehyde (MDA) and the antioxidant marker superoxide dismutase (SOD) were determined in the livers. As shown in Figure 3, the content of MDA in the liver tissues was significantly reduced, and SOD activity was enhanced in the HIR PKC θ deletion mice when compared to the HIR WT mice in the liver. Moreover, heme treatment decreased the MDA level, and increased SOD activity in the WT mice compared with untreated WT mice in the liver. Treatment with heme also lowered the content of MDA and raised SOD activity in the HIR PKC θ deletion mice when compared to the untreated PKC θ deletion mice and the heme-treated WT mice.

To further discuss the mechanism of PKC θ deletion on pivotal antioxidant defense regulators in HIP injury, the expression of HO-1 and Nrf2 was evaluated (Figure 3C). The results demonstrated that PKC θ deletion markedly elevated the HO-1 (Figure 3D) and Nrf2 (Figure 3E) protein expression compared to the WT mice, heme treatment was also observed to elevate the HO-1 and Nrf2 protein expression compared with the untreated PKC θ deletion mice and the heme-treated WT mice (Figure 3D, E).

***Inflammatory cytokines***

**Figure 4:**
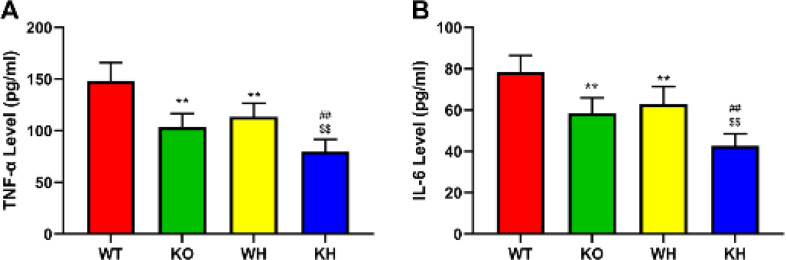
Effect of PKC-θ knockout on inflammatory factors. (A) TNF α level in serum. (B) IL-6 level in serum

Inflammation has been demonstrated to play a pivotal effect in the development of HIR injury (19). Therefore, the proinflammatory factors, including TNF-α and IL-6, were determined to estimate if PKC θ deletion attenuates inflammatory response induced by HIR. Our results showed that PKC θ deletion reduced serum TNF-α and IL-6 levels induced by HIR compared to the WT mice (Figure 4). Treatment with heme significantly decreased the serum TNF-α and IL-6 levels in the HIR PKC **θ** deletion mice compared with the untreated PKC θ deletion mice and the heme-treated WT mice (Figure 4).

***Histopathological assessment***

**Figure 5:**
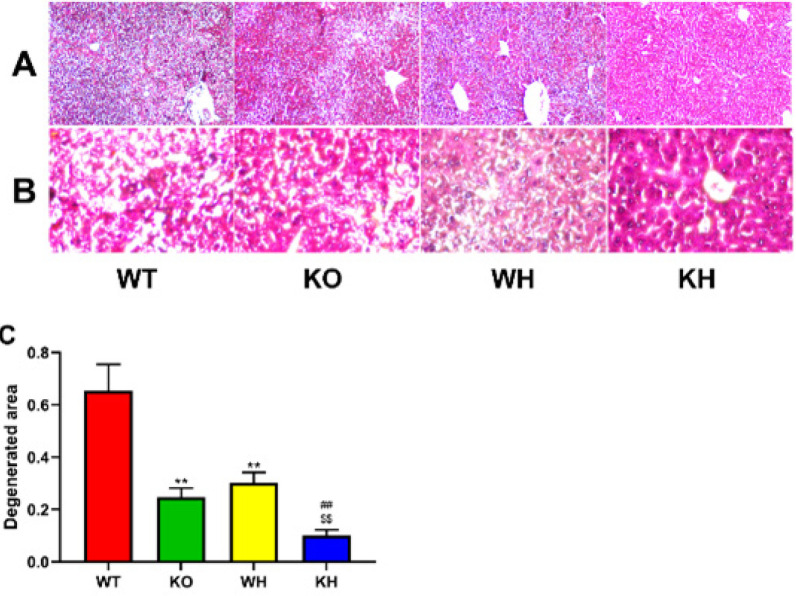
Morphological changes in the liver. (A) Liver tissues stained with HE (100×). (B) Liver tissues stained with HE (400×). (C) Degenerated area of the liver

The histopathological examination of the liver of the HIR WT mice showed a poor hepatic architecture with swollen hepatocytes, disorder cords, and dilated sinusoids with vacuolation, together with focal pale eosinophilic area. Inflammatory cell infiltration and severe dilation and congestion of the central vein were observed in the liver of the HIR WT mice. Impairment of hepatic architecture was attenuated in the PKC θ deletion mice and the treated WT mice with heme when compared to the HIR WT mice. Additionally, dilation and congestion of the central vein in the livers were reduced in the PKC θ deletion mice and the treated WT mice with heme. Furthermore, the histopathological lesions were markedly regressed when compared to the PKC θ deletion mice, and the central vein was lightly dilated and congested (Figure 5A, B).

The degenerated areas within the hepatic lobules showed a significant decrease in the PKC θ deletion mice when compared to the WT mice (Figure 5C). In addition, HIR PKC θ deletion mice treatment with heme markedly decreased the degenerated areas compared with the untreated PKC θ deletion mice and the heme-treated WT mice with heme (Figure 5C).

***Immunofluorescence analysis***

**Figure 6:**
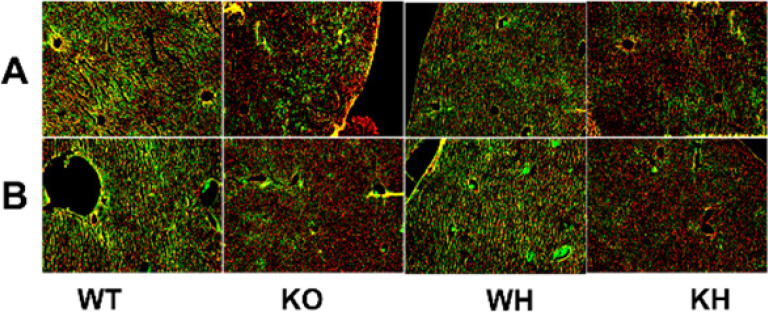
Immunofluorometric analysis. (A) NF-κB immunofluorescence. (B) Caspase 3 immunofluorescence

WT: wild-type mice; KO: PKC-θ knockout mice; WH: wild-type mice treated with heme; KH: PKC-θ knockout mice treated with heme

Immunofluorescence results showed that more staining spots for NF-κB and caspase 3 were observed in the HIR WT mice than in the PKC θ deletion mice and the treated WT mice with heme. Meanwhile, Treatment with heme reduced staining spots for NF-κB and caspase 3 staining in the liver section of the PKC θ deletion mice when compared to the untreated gene deletion mice and the WT mice treated with heme. The findings suggested that the gene deletion attenuated the HIR injury by elevating the anti-inflammatory effect and antioxidation (Figure 6).

***Effect of PKC θ knockout on related protein expression***

**Figure 7:**
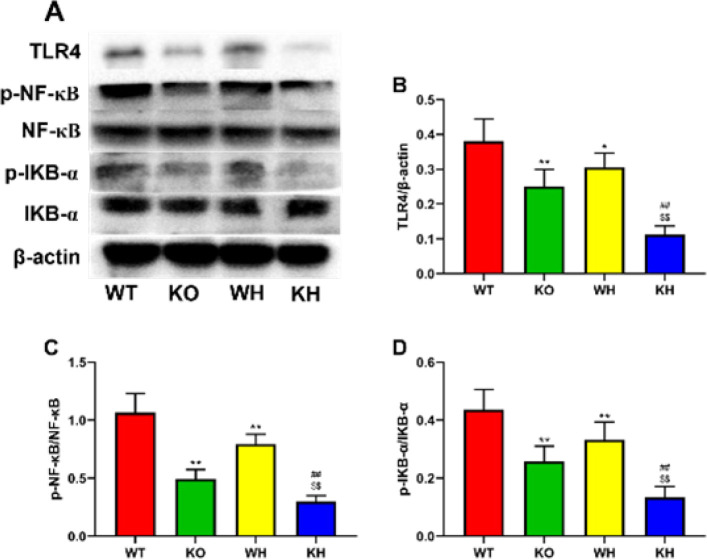
Effect of PKC-θ knockout on protein expression. (A) Expression of TLR4, p-NF κB, and p-IκB α in the liver. (B) Relative level of TLR 4. (C) Relative level of p-NF κB. (D) Relative level of p-IκB α

PKC θ deletion markedly decreased the expressions of TLR4 and phosphorylated NF-κB and IKB-α when compared to the WT mice (Figure 7). Treatment with heme to PKC θ deletion mice further decreased the expressions of TLR4 and phosphorylated NF-κB and IKB-α compared with the untreated knockout mice and treated WT mice with heme (Figure 7).

**P-value<0.01 compared with WT mice; ^$$^ *P-value<*0.01 compared with KO mice; ^##^*P-value*<0.01 compared with WH mice

ALT: alanine aminotransferase; AST: aspartate aminotransferase; WT: wild type mice; KO: PKC-θ knockout mice; WH: wild type mice treated with heme; KH: PKC-θ knockout mice treated with heme

**P-value<0.01 compared with WT mice; ^$$^ *P-value<*0.01 compared with KO mice; ^##^* P-value<*0.01 compared with WH mice

WT: wild-type mice; KO: PKC-θ knockout mice; WH: wild-type mice treated with heme; KH: PKC-θ knockout mice treated with heme

**P-value<0.01 compared with WT mice; ^$$ ^*P-value<*0.01 compared with KO mice; ^##^ *P-value<*0.01 compared with WH mice

WT: wild-type mice; KO: PKC-θ knockout mice; WH: wild-type mice treated with heme; KH: PKC-θ knockout mice treated with heme

**P-value<0.01 compared with WT mice; ^$$^ *P-value<*0.01 compared with KO mice; ^##^*P-value<*0.01 compared with WH mice

WT: wild-type mice; KO: PKC-θ knockout mice; WH: wild-type mice treated with heme; KH: PKC-θ knockout mice treated with heme

*P-value<0.05, **P-value<0.01 compared with WT mice; ^$$ ^*P-value<*0.01 compared with KO mice; ^## ^*P-value<*0.01 compared with WH mice

WT: wild-type mice; KO: PKC-θ knockout mice; WH: wild-type mice treated with heme; KH: PKC-θ knockout mice treated with heme

## Discussion

HIR injury is a complicated fatal process in pathophysiology and results from the interruption of blood flow in the liver, following restoration, which leads to hepatic damage and hepatocyte death (20, 21). At present, there are few effective strategies for the treatment of HIR injury due to lacking mechanisms responsible for HIR injury (22). In the current study, we investigated the effect of PKC θ deletion on HIR injury and its possible mechanism. The results from the current study showed that PKC θ deletion reduced the hepatic function marker serum ALT and AST levels, attenuated hepatic injury, elevated the SOD activity and the Nrf2 and HO-1 protein expression, and reduced MDA content, serum levels of TNF-α, and IL-6 when compared to the WT mice. Additionally, PKC θ deletion decreased the expressions of TLR4 and phosphorylated NF-κB and IKB-α in the liver. Furthermore, PKC θ deletion mice heme treatment markedly decreased the serum levels of ALT and AST, elevated antioxidation and alleviated inflammation, and improved Nrf2/HO-1 and TLR4/NF-κB/IKB-α pathways compared with the untreated PKC θ deletion and the treated WT mice.

In the current study, a suitable HIR mice model in the PKC θ deletion and WT mice was used in the experiment. Induction of HIR in the PKC θ deletion mice caused a reduction in hepatic function markers ALT and AST and reduced degenerated area when compared to the WT mice, suggesting that gene deletion attenuated acute liver injury. Meanwhile, heme pretreatment significantly decreased the ALT and AST levels in the serum of the PKC θ deletion mice compared with the untreated gene deletion mice, together with the degenerated area of the liver. Additionally, heme treatment also reduced the serum ALT and AST levels and the degenerated area in the WT mice. PKC θ deletion mice treated with or without heme were also found to improve the structure of the liver damage induced by IR. These results pointed to the protective effect of PKC θ knockout and/or heme treatment against HIR injury.

Oxidative stress caused by excessive ROS and depletion of the antioxidant defense system plays a pivotal role in IR injury (23, 24). Some experiments have demonstrated that the restoration of blood supply following hepatic ischemia results in excessive production of ROS and disorder of antioxidant scavengers, which promotes lipid peroxidation, and DNA injury, damaging the cell membranes and causing inflammation, and even cell death (25-27). MDA is an important biomarker of lipid peroxidation, and superoxide scavenger SOD, an antioxidant metalloenzyme, exerts a crucial effect in the defense system against oxidative stress (28, 29). The findings from the current study confirmed that PKC θ deletion markedly lowered MDA content and elevated SOD activity in ischemia/reperfusion liver when compared to the WT mice. In addition, PKC θ deletion elevated the Nrf2 and HO-1 protein expression. Previous reports showed that inhibition of PKC θ reduced the level of ROS (30). These results suggest that reducing gene deletion can improve oxidative stress associated with HIR injury.

HO-1, a widely expressed inducible enzyme, can transform heme into bilirubin, carbon monoxide (CO), and ferrous iron (31), The metabolites of heme, including CO, bilirubin, and Fe^2+^, can scavenge free radicals, singlet oxygen, and superoxide anions, and exert anti-inflammation, antioxidation, and anti-apoptosis and attenuate oxidative stress-induced tissue damages including HIR injury (32-35), HO-1 induced by heme exhibits multifunctional effects. It has been demonstrated that HO-1 exerts the prevention of carcinogenesis via maintaining redox homeostasis (36). Recent studies demonstrated that HO-1 attenuates testicular ischemia/reperfusion injury (37), improves myelodysplastic syndromes (MDSs)(38), and protects the remnant liver against dysfunction after major hepatectomy (39). It has been confirmed that nuclear factor-erythroid 2 p45-related factor 2 (Nrf2) is involved in the regulation of HO-1 expression. Nrf2, a pivotal transcription factor, plays a pivotal effect in mediating the transcription and the expression of antioxidant enzymes, exerting a protective effect against various stress-induced damages (40). The effect of the Nrf2/HO-1 signaling on oxidative stress diseases is associated with the regulation of antioxidants (41). To further explore the mechanism of PKC θ in HIR injury, HO-1 was induced in the WT and PKC θ deletion mice with heme, and the animals were used to prepare the model of HIR. The data from the study suggested that PKC θ deletion elevated the HO-1 and Nrf2 protein expressions in the HIR PKC θ deletion mice when compared to the WT mice. Treatment with heme in mice increased the HO-1 and Nrf2 protein expressions compared with the untreated mice. Additionally, HO-1 and Nrf2 expressions were increased in the HIR gene deletion mice when compared to the WT mice. The findings showed that up-regulation of the Nrf2/ HO-1 pathway was implicated in the attenuation of HIR injury by PKC θ deletion.

Inflammatory responses have been confirmed to contribute to HIR injury (42). Some studies indicated that inflammatory cells are activated in the HIR injury, triggering the generation of pro-inflammatory factors including IL-6 and TNF α (43, 44). TNF α stimulates the expression of various adhesion molecules and chemokines, while resulting in the excessive production of ROS and proteases, causing severe tissue damage (45). Toll-like receptor 4 (TLR4) has been demonstrated to exert an important effect on the production of inflammatory cytokines after IR (46, 47). Further study demonstrated that TLR4 regulates the release of pro-inflammatory factors via regulation of nuclear factor kappa B (NF κB) activation (48). The TLR4/NF-κB signaling pathway was found to regulate inflammation in ischemic tissue injury such as the heart, liver, and lung (49-51). NF-κB bound to the inhibitor IκB α covers the nuclear localization sites, inhibiting the NF-κB translocation to the cell nucleus. Activation of NF-κB increases the translocation of NF-κB to the nucleus, mediating the inflammatory factor expressions (42). PKC-θ, a novel calcium-independent PKC isoenzyme, highly expressed in T cells, is involved in the modulation of cell proliferation and survival (52). Additionally, it has been confirmed that PKC-θ is involved in the mediation of muscle development and homeostasis and is also associated with the modulation of platelet activation, aggregation, and hemostasis (53, 54). It has been demonstrated that PKC-θ can activate NF κB and activator protein 1(AP-1), regulating the cytokine expression (55). The data from the study indicated that gene deletion decreased serum TNF α and IL-6 levels and the expression of TLR4, phosphorylated NF κB, and IKB α in the liver when compared to the WT mice. Induction of HO-1 by heme was observed to significantly improve serum TNF α and IL-6 levels and the expression of TLR4 phosphorylated NF κB and IKB α when compared to the untreated gene deletion and the treated WT mice. HO-1 was found to exert its beneficial role via regulation of inflammation and oxidative stress (56). These results suggested that the protective effect of PKC-θ deletion against HIR injury is associated with the TLR4/NF-κB pathway. 

Furthermore, the findings from the present study were applied in the teaching of pathophysiology, strengthening the understanding of our colleagues and students involved in the study on the pathophysiological mechanism of PKC-θ deletion in HIR injury and students’ innovative thinking, and widening students’ academic horizons.

## Conclusion

Taken together, the results from this study demonstrated that PKC-θ knockout was able to improve the liver function and the structure of the impaired liver induced by IR and attenuate HIR injury. This beneficial effect was related to, at least in part, the improvement of the antioxidative effect via regulation of Nrf2/HO-1 signaling and enhancement of the anti-inflammatory effect by down-regulating the TLR4/ NF-κB/IKB α signaling. These findings may provide a therapeutic target of the drug for HIR injury.

## References

[1] Simillis C, Robertson FP, Afxentiou T, Davidson BR, Gurusamy KS (2016). A network meta-analysis comparing perioperative outcomes of interventions aiming to decrease ischemia reperfusion injury during elective liver resection. Surgery.

[2] Gendy A, Elnagar MR, Soubh A, Al-Mokaddem A, El-Haddad A, El-Sayed MK (2021). Morin alleviates hepatic ischemia/reperfusion-induced mischief: In vivo and in silico contribution of Nrf2, TLR4, and NLRP3. Biomed Pharmacother.

[3] Li J, Li RJ, Lv GY, Liu HQ (2015). The mechanisms and strategies to protect from hepatic ischemia-reperfusion injury. Eur Rev Med Pharmacol Sci.

[4] Dickson I (2019). Improving hepatic ischaemia-reperfusion injury outcomes. Nat Rev Gastroenterol Hepatol.

[5] Huang M, Cai H, Han B, Xia YH, Kong XN, Gu JY (2022). Natural Killer Cells in Hepatic Ischemia-Reperfusion Injury. Front Immunol.

[6] He ZG, Ma C, Yu TY, Song J, Leng J, Gu XB (2019). Activation mechanisms and multifaceted effects of mast cells in ischemia reperfusion injury. Exp Cell Res.

[7] Song X, Qian XQ, Shen M, Jiang R, Wagner MB, Ding GL (2015). Protein kinase C promotes cardiac fibrosis and heart failure by modulating galectin-3 expression. Biochim Biophys Acta Mol Cell Res.

[8] Newton AC (2018). Protein kinase C: Perfectly balanced. Crit Rev Biochem Mol Biol.

[9] Marrocco V, Bogomolovas J, Ehler E, dos Remedios CG, Yu JY, Gao C (2019). PKC and PKN in heart disease. J Mol Cell Cardiol.

[10] Gào X, Schöttker B (2017). Reduction-oxidation pathways involved in cancer development: a systematic review of literature reviews. Oncotarget.

[11] Wang XP, Yao Y, Li YX, Guo SJ, Li YJ, Zhang GQ (2023). Experimental study on the effect of luteolin on the proliferation, apoptosis and expression of inflammation-related mediators in lipopolysaccharide-induced keratinocytes. Int J Immunopathol Pharmacol.

[12] Madouri F, Chenuet P, Beuraud C, Fauconnier L, Marchiol T, Rouxel N (2017). Protein kinase Cθ controls type 2 innate lymphoid cell and T2 responses to house dust mite allergen. J Allergy Clin Immunol.

[13] Fiore PF, Benedetti A, Sandonà M, Madaro L, De Bardi M, Saccone V (2020). Lack of PKCθ promotes regenerative ability of muscle stem cells in chronic muscle injury. Int J Mol Sci.

[14] Song YN, Lee JW, Ryu HW, Lee JK, Oh ES, Kim DY (2023). Black ginseng extract exerts potentially anti-asthmatic activity by inhibiting the protein kinase Cθ-mediated IL-4/STAT6 signaling pathway. Int J Mol Sci.

[15] Jiang L, Chi CH, Yuan F, Lu MQ, Hu DQ, Wang L (2022). Anti-inflammatory effects of anemonin on acute ulcerative colitis via targeted regulation of protein kinase C-θ. Chin Med.

[16] Alharshawi K, Marinelarena A, Kumar P, El-Sayed O, Bhattacharya P, Sun Z (2017). PKC-ѳ is dispensable for OX40L-induced TCR-independent Treg proliferation but contributes by enabling IL-2 production from effector T-cells. Sci Rep.

[17] Pu JL, Huang ZT, Luo YH, Mou T, Li TT, Li ZT (2021). Fisetin mitigates hepatic ischemia-reperfusion injury by regulating GSK3β/AMPK/NLRP3 inflammasome pathway. Hepatobiliary Pancreat Dis Int.

[18] Wang GG, Lu XH, Ding M, Tang WT, Li W, Zhao X (2011). [Protective effects of luteolin preconditioning on rat liver under ischemia/reperfusion]. Sheng Li Xue Bao.

[19] Yu HC, Bai L, Yang ZX, Qin HY, Tao KS, Han H (2016). Blocking Notch signal in myeloid cells alleviates hepatic ischemia reperfusion injury by repressing the activation of NF-κB through CYLD. Sci Rep.

[20] Wang YC, Yang Y, Wang M, Wang SH, Jeong JM, Xu L (2021). Eosinophils attenuate hepatic ischemia-reperfusion injury in mice through ST2-dependent IL-13 production. Sci Transl Med.

[21] Ito Y, Hosono K, Amano H (2023). Responses of hepatic sinusoidal cells to liver ischemia-reperfusion injury. Front Cell Dev Biol.

[22] Ahmed O, Robinson MW, O’Farrelly C (2021). Inflammatory processes in the liver: Divergent roles in homeostasis and pathology. Cell Mol Immunol.

[23] Jiang Y, He XX, Simonaro CM, Yi B, Schuchman EH (2021). Acid ceramidase protects against hepatic ischemia/reperfusion injury by modulating sphingolipid metabolism and reducing inflammation and oxidative stress. Front Cell Dev Biol.

[24] Li ZX, Wang Y, Zhang Y, Wang X, Gao BQ, Li Y (2021). Protective effects of fisetin on hepatic ischemia-reperfusion injury through alleviation of apoptosis and oxidative stress. Arch Med Res.

[25] Zhu CL, Shi SH, Jiang P, Huang XJ, Zhao JX, Jin Y (2023). Curcumin alleviates hepatic ischemia-reperfusion injury by inhibiting neutrophil extracellular traps formation. J Invest Surg.

[26] Yang H, Huang ZT, Luo YH, Lei DL, Yan P, Shen A (2023). TRIM37 exacerbates hepatic ischemia/reperfusion injury by facilitating IKKγ translocation. Mol Med.

[27] Cadenas S (2018). ROS and redox signaling in myocardial ischemia-reperfusion injury and cardioprotection. Free Rad Biol Med.

[28] Catanzaro R, Zerbinati N, Solimene U, Marcellino M, Mohania D, Italia A (2016). Beneficial effect of refined red palm oil on lipid peroxidation and monocyte tissue factor in HCV-related liver disease: a randomized controlled study. Hepatobiliary Pancreat Dis Int.

[29] Xu GP, Wang XL, Xiong YX, Ma XP, Qu L (2019). Effect of sevoflurane pretreatment in relieving liver ischemia/reperfusion-induced pulmonary and hepatic injury. Acta Cir Bras.

[30] Kammouni W, Wood H, Jackson AC (2017). Lyssavirus phosphoproteins increase mitochondrial complex I activity and levels of reactive oxygen species. J Neurovirol.

[31] Zhang XY, Yu YH, Lei HY, Cai YF, Shen J, Zhu P (2020). The Nrf-2/HO-1 signaling axis: A ray of hope in cardiovascular diseases. Cardiol Res Pract.

[32] Yu QW, Chen SY, Tang HW, Zhang XD, Tao RL, Yan ZP (2021). Veratric acid alleviates liver ischemia/reperfusion injury by activating the Nrf2 signaling pathway. Int Immunopharmacol.

[33] Zhao Y, Kong GY, Pei WM, Zhou B, Zhang QQ, Pan BB (2019). Dexmedetomidine alleviates hepatic injury via the inhibition of oxidative stress and activation of the Nrf2/HO-1 signaling pathway. Eur Cytokine Netw.

[34] Chen QY, Wang GG, Li W, Jiang YX, Lu XH, Zhou PP (2016). Heme oxygenase-1 promotes delayed wound healing in diabetic rats. J Diabetes Res.

[35] Bao LP, Li JS, Zha DQ, Zhang L, Gao P, Yao T (2018). Chlorogenic acid prevents diabetic nephropathy by inhibiting oxidative stress and inflammation through modulation of the Nrf2/HO-1 and NF-κB pathways. Int Immunopharmacol.

[36] Nitti M, Ivaldo C, Traverso N, Furfaro AL (2021). Clinical significance of heme oxygenase 1 in tumor progression. Antioxidants.

[37] Xie B, Cheng B, He L, Liu Y, He N (2024). HO-1 attenuates testicular ischaemia/reperfusion injury by activating the phosphorylated C-jun-miR-221/222-TOX pathway. Heliyon.

[38] Sadeghi M, Fathi M, Navashenaq JG, Mohammadi H, Yousefi M, Hojjat-Farsangi M (2023). The prognostic and therapeutic potential of HO-1 in leukemia and MDS. Cell Commun Signal.

[39] He N, Sun XN, Hu ZH, Wang FF, Zhang Y, Chen XW (2022). HO-1 protects remnant liver against dysfunction after major hepatectomy in humans. J Investig Surg.

[40] Yang WC, Wang YX, Zhang CG, Huang YZ, Yu JX, Shi L (2022). Maresin1 protect against ferroptosis-induced liver injury through ROS inhibition and Nrf2/HO-1/GPX4 activation. Front Pharmacol.

[41] Zhang XY, Ding M, Zhu P, Huang HL, Zhuang Q, Shen J (2019). New insights into the Nrf-2/HO-1 signaling axis and its application in pediatric respiratory diseases. Oxid Med Cell Longev.

[42] Yoon SH, Kang HB, Kim J, Yoo K, Han SJ (2022). Diminazene aceturate attenuates hepatic ischemia/reperfusion injury in mice. Sci Rep.

[43] Kaltenmeier C, Wang RH, Popp B, Geller D, Tohme S, Yazdani HO (2022). Role of immuno-inflammatory signals in liver ischemia-reperfusion injury. Cells.

[44] Ni M, Zhang J, Sosa R, Kageyama S, Busuttil R, Elaine R (2021). TIM-4+kupffer cells play homeostatic roles in liver ischemia reperfusion injury. Hepatology.

[45] Chen D, Wei L, Chen Z (2017). Prevention of aged liver ischemia reperfusion injury by silencing the expression of liver TNF-α and complement 3 gene in mice. Transplantation.

[46] Yang RH, Song ZX, Wu SQ, Wei Z, Xu Y, Shen XC (2018). Toll-like receptor 4 contributes to a myofibroblast phenotype in cardiac fibroblasts and is associated with autophagy after myocardial infarction in a mouse model. Atherosclerosis.

[47] Morsy MA, Ibrahim YF, Hafez SMNA, Zenhom NM, Nair AB, Venugopala KN (2022). Paeonol attenuates hepatic ischemia/reperfusion injury by modulating the Nrf2/HO-1 and TLR4/MYD88/NF-κB signaling pathways. Antioxidants.

[48] Hu XJ, Ding CG, Ding XM, Fan P, Zheng J, Xiang HL (2020). Inhibition of myeloid differentiation protein 2 attenuates renal ischemia/reperfusion-induced oxidative stress and inflammation via suppressing TLR4/TRAF6/NF-kB pathway. Life Sci.

[49] Morsy MA, Abdel-Gaber SA, Rifaai RA, Mohammed MM, Nair AB, Abdelzaher WY (2022). Protective mechanisms of telmisartan against hepatic ischemia/reperfusion injury in rats may involve PPARγ-induced TLR4/NF-κB suppression. Biomed Pharmacother.

[50] Zhang X, Du QM, Yang Y, Wang JN, Dou S, Liu C (2017). The protective effect of Luteolin on myocardial ischemia/reperfusion (I/R) injury through TLR4/NF-κB/NLRP3 inflammasome pathway. Biomed Pharmacother.

[51] Yu Z, Tong Y, Zhang RLZ, Ding XB, Li Q (2017). Saquinavir ameliorates liver warm ischemia-reperfusion-induced lung injury via HMGB-1-and P38/JNK-mediated TLR-4-dependent signaling pathways. Mediators Inflamm.

[52] Anto NP, Muraleedharan A, Nath PR, Sun ZM, Keasar C, Livneh E (2023). The Peptidyl-Prolyl isomerase, Pin1, associates with Protein Kinase C ? via a critical Phospho-Thr-Pro motif in the V3 regulatory domain. Front Immunol.

[53] Al-Sabri MH, Behare N, Alsehli AM, Berkins S, Arora A, Antoniou E (2022). Statins induce locomotion and muscular phenotypes in that are reminiscent of human myopathy: Evidence for the role of the chloride channel inhibition in the muscular phenotypes. Cells.

[54] Zaid Y, Senhaji N, Naya A, Fadainia C, Kojok K (2015). PKCs in thrombus formation. Pathol Biol.

[55] Lim PS, Sutton CR, Rao S (2015). Protein kinase C in the immune system: from signalling to chromatin regulation. Immunology.

[56] Xu C, Song YL, Wang ZG, Jiang JZ, Piao YH, Li L (2021). Pterostilbene suppresses oxidative stress and allergic airway inflammation through AMPK/Sirt1 and Nrf2/HO-1 pathways. Immun Inflamm Dis.

